# Genetic identification and evolutionary trends of the seagrass *Halophila nipponica* in temperate coastal waters of Korea

**DOI:** 10.1371/journal.pone.0177772

**Published:** 2017-05-15

**Authors:** Young Kyun Kim, Seung Hyeon Kim, Joo Mi Yi, Chang-Keun Kang, Frederick Short, Kun-Seop Lee

**Affiliations:** 1 Department of Biological Sciences, Pusan National University, Busan, Republic of Korea; 2 School of Earth Sciences and Environmental Engineering, Gwangju Institute of Science and Technology, Gwangju, Republic of Korea; 3 Research Center, Dongnam Institute of Radiological & Medical Sciences (DIRAMS), Busan, Republic of Korea; 4 Jackson Estuarine Laboratory, University of New Hampshire, Durham, New Hampshire, United States of America; Saint Mary's University, CANADA

## Abstract

Although seagrass species in the genus *Halophila* are generally distributed in tropical or subtropical regions, *H*. *nipponica* has been reported to occur in temperate coastal waters of the northwestern Pacific. Because *H*. *nipponica* occurs only in the warm temperate areas influenced by the Kuroshio Current and shows a tropical seasonal growth pattern, such as severely restricted growth in low water temperatures, it was hypothesized that this temperate *Halophila* species diverged from tropical species in the relatively recent evolutionary past. We used a phylogenetic analysis of internal transcribed spacer (ITS) regions to examine the genetic variability and evolutionary trend of *H*. *nipponica*. ITS sequences of *H*. *nipponica* from various locations in Korea and Japan were identical or showed very low sequence divergence (less than 3-base pair, bp, difference), confirming that *H*. *nipponica* from Japan and Korea are the same species. *Halophila* species in the section *Halophila*, which have simple phyllotaxy (a pair of petiolate leaves at the rhizome node), were separated into five well-supported clades by maximum parsimony analysis. *H*. *nipponica* grouped with *H*. *okinawensis* and *H*. *gaudichaudii* from the subtropical regions in the same clade, the latter two species having quite low ITS sequence divergence from *H*. *nipponica* (7–15-bp). *H*. *nipponica* in Clade I diverged 2.95 ± 1.08 million years ago from species in Clade II, which includes *H*. *ovalis*. According to geographical distribution and genetic similarity, *H*. *nipponica* appears to have diverged from a tropical species like *H*. *ovalis* and adapted to warm temperate environments. The results of divergence time estimates suggest that the temperate *H*. *nipponica* is an older species than the subtropical *H*. *okinawensis* and *H*. *gaudichaudii* and they may have different evolutionary histories.

## Introduction

Seagrasses are a polyphyletic group of monocotyledonous angiosperms that evolved in the marine environment approximately 100 million years ago (Mya) [1–4]. Seagrasses have adapted successfully to marine environments and play important roles in coastal and estuarine ecosystems, providing food, habitat, and shelter to a wide variety of marine animals [5–7]. Although seagrasses are distributed in nearly all coastal areas of the world, the global species diversity of seagrasses is extremely low (approximately 72 species) compared to terrestrial angiosperms [8–10].

Seagrasses can adapt to either tropical or temperate thermal regimes. There is roughly the same number of temperate and tropical genera and species, while a few genera and species occur in both climate zones [9]. The marine members of the family Hydrocharitaceae, which includes the seagrass genera *Enhalus*, *Thalassia*, and *Halophila*, do not usually occur where minimum temperatures are less than 20°C [11,12], and consequently the genera in this family have been considered largely tropical [9,13]. Although most *Halophila* species occur in tropical or subtropical regions, some *Halophila* species such as *H*. *australis* are distributed in temperate regions [13]. *H*. *nipponica* also occurs in warm temperate areas of the northwestern Pacific [14–17].

*H*. *nipponica* was first observed in temperate regions of the Japanese archipelago approximately 100 years ago, and was subsequently treated as *H*. *ovalis* because species classification and identification in the genus *Halophila* are controversial due to morphological similarity and variability [1,12,14,18]. This temperate *Halophila* species was described as a new species, *H*. *nipponica*, in 2006 based on morphology [14]. In the same year, *H*. *japonica* was described using both morphology and genetics [19]; subsequently, *H*. *japonica* was treated as a synonym of *H*. *nipponica* [15]. The first observation of *H*. *nipponica* on the southern coast of the Korean peninsula occurred in 2007, with its identification based on morphology [16]. Since then, many meadows of *H*. *nipponica* have been observed along the warm southern coast of Korea [17]. *H*. *nipponica* is now known to be widely distributed in warm temperate Korean and Japanese waters, and considered endemic to Korea and Japan [15,16]. Since *H*. *nipponica* occurs in temperate coastal waters of the northwestern Pacific, but is not present in the tropical west Pacific and Indian Oceans, this species has been considered a temperate-adapted *Halophila* species [14,16]. However, growth of *H*. *nipponica* is minimal at water temperature less than 15°C, and no growth reduction in high summer water temperature was observed, implying that this species still possess a tropical seasonal growth pattern [17].

The genus *Halophila* contains approximately 20 species and consists of five sections, a taxonomic rank between the genus and the species, based on morphological differences [10,13,14]. Most species in the genus *Halophila* are in section *Halophila*, which contains species with a pair of petiolate leaves borne on short erect lateral shoots [13,14]. All other species are in the sections *Microhalophila* (*H*. *beccarii*), *Spinulosae* (*H*. *spinulosa*), *Tricostata* (*H*. *tricostata*), and *Americanae* (*H*. *engelmannii* and *H*. *baillonis*) [13]. Although identification of *Halophila* species has been established by various taxonomic studies [14], molecular genetic studies proposed that *Halophila* species such as *H*. *johnsonii* and *H*. *hawaiiana* should be treated as conspecific with *H*. *ovalis* [20,21]. Therefore, further taxonomic and molecular genetic studies are required for more accurate species classification of the genus *Halophila*.

The internal transcribed spacer (ITS) region of nuclear ribosomal DNA (nrDNA) is phylogenetically informative and useful in understanding the evolutionary and biogeographic relationships among closely related taxa [3,15,20–25]. DNA sequences of the ITS region evolve rapidly and may vary among species within a genus or among populations of the same species [23,24,26,27]. Phylogenetic analyses of the ITS region of nrDNA have been conducted to investigate the taxonomic status and to infer biogeographic trends in the genus *Halophila*, suggesting that a molecular phylogenetic study of this region is useful to differentiate major taxonomic groups within the genus *Halophila* [3,15,20,21,25]. In this study, we conducted a phylogenetic analysis of the ITS region of nrDNA to assess genetic variability among *H*. *nipponica* collected from populations across its known range in Korean and Japanese coastal waters and to elucidate position of the species *H*. *nipponica* among the taxonomic groups of the genus *Halophila*. The evolutionary trend of *H*. *nipponica* was inferred using DNA sequences of the ITS region from all assessed *Halophila* species.

## Materials and methods

### Locations of *Halophila nipponica* and plant sample collection

**Fig 1:**
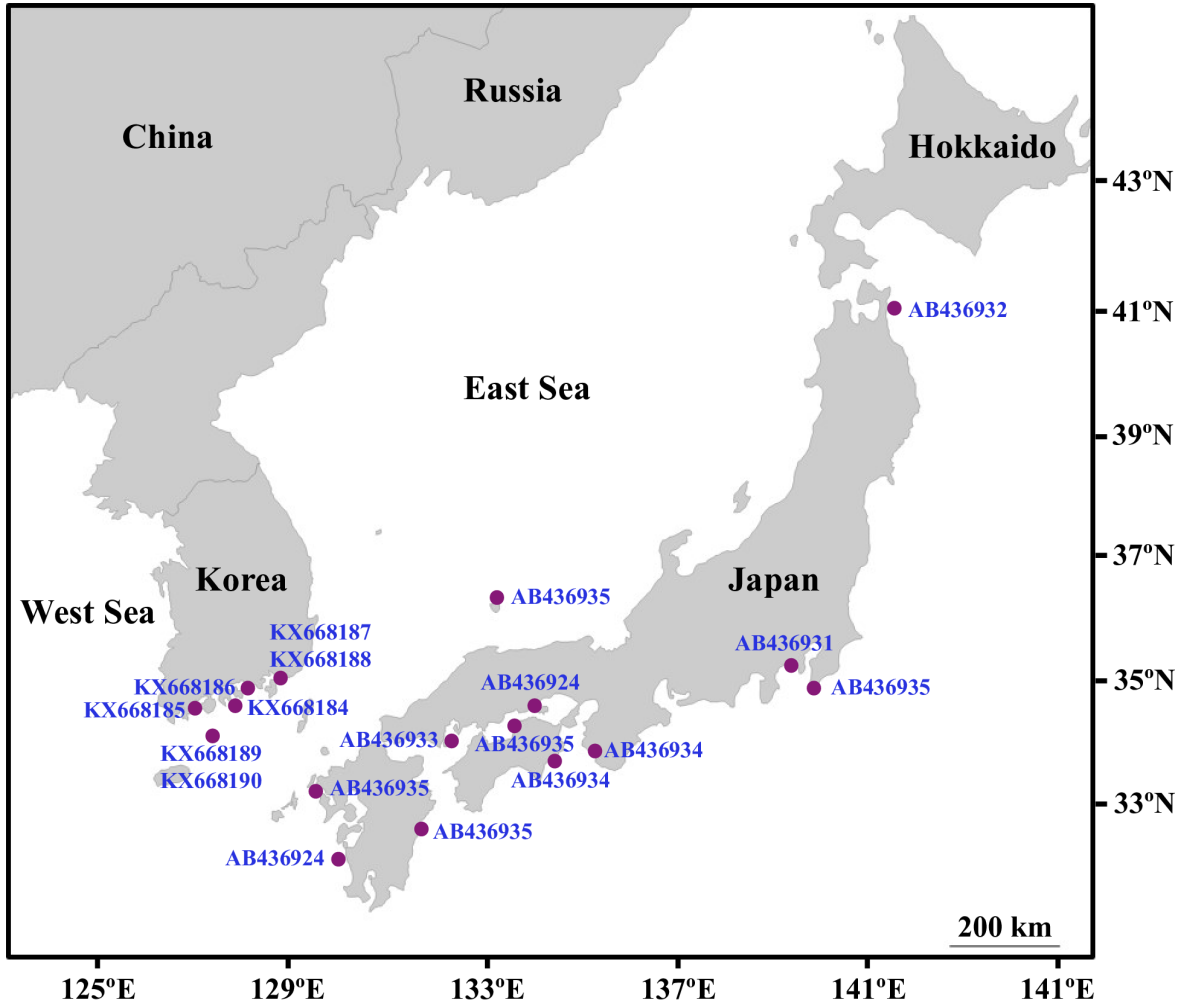
*Halophila nipponica* populations in the temperate waters of Korea and Japan. Geographical locations for *H*. *nipponica* populations in Japanese waters were obtained from Uchimura et al. [15].

*H*. *nipponica* is found on the southern coast of Korea and in temperate coastal waters of the Japanese archipelago with the exception of Hokkaido, the northernmost island of Japan (Fig 1). *H*. *nipponica* shoots were collected for DNA extraction from five seagrass meadows on the southern coast of Korea at 5-m intervals using SCUBA (Fig 1). Fifty-four *H*. *nipponica* samples, including 20 from An-do Island (HN01–HN20), 8 from Sorok-do Island (HN21–HN28), 12 from Namhae Island (HN31–HN42), 10 from Koje Island (HN51–HN60), and 4 from Geomun-do Island (HN61–HN64), were collected for sequencing of ITS regions (Table 1). Plant samples of *H*. *ovalis* (n = 3) and *H*. *minor* (n = 3) were collected in Trang, Thailand. No specific permissions to collect research samples were required at the study sites, and the field study did not involve endangered or protected species. After collection, samples were cleaned with distilled water, desiccated, and stored at room temperature in silica gel for later DNA extraction. Portions of each collection were preserved as herbarium voucher specimens, and deposited in the lab and the Herbarium of Kyungpook National University.

**Table 1:** Collection information and GenBank accession numbers of *Halophila nipponica* specimens used for phylogenetic analysis from various geographical locations in the temperate coastal waters of northeast Asia.

Species	Collection information	Depth (m)	N	Sample label	Herbarium no.	GenBank accession no.
***Halophila nipponica***	An-do Island (34°28′48″N, 128°48′35″E), Korea	6	20	HN01–HN20	*Kim* 2009030501–3 (KNU)	KX668184
***Halophila nipponica***	Sorok-do Island (34°31′01″N, 127°08′13″E), Korea	4	8	HN21–HN28		KX668185
***Halophila nipponica***	Namhae Island (34°43′39″N, 128°02′11″E), Korea	4–8	12	HN31–HN42		KX668186
***Halophila nipponica***	Koje Island (34°57′47″N, 128°37′19″E), Korea	3	10	HN51–55, HN58–60		KX668187
				HN56, 57		KX668188
***Halophila nipponica***	Geomun-do Island (34°03′21″N, 127°16′58″E), Korea	8	4	HN61, 63, 64		KX668189
				HN62		KX668190
***Halophila nipponica***	Odawa Bay (35°13′16″N, 139°37′17″E), Japan	-	-	-	TNS 753656	AB436931
***Halophila nipponica***	Mutsu Bay (41°00′36″N, 140°40′01″E), Japan	-	-	-	TNS 753676	AB436932
***Halophila nipponica***	Suou-Oohshima, Yamaguchi Pref., Japan	-	-	-	TNS 753649	AB436933
***Halophila nipponica***	Mugi-Ooshima (33°38′10″N, 134°29′13″E), Japan	-	-	-	TNS 753651	AB436934
	Ezura,Wakayama Pref., Japan	-	-	-	CBM 238518	AB436934
***Halophila nipponica***	Urumi (36°01′49″N, 133°01′29″E), Japan	-	-	-	TNS 753668	AB436935
	Oohama (34°13′33″N, 133°36′35″E), Japan	-	-	-	TNS 753671	AB436935
	Mihokogaura (32°25′19″N, 131°40′30″E), Japan	-	-	-	TNS 753672	AB436935
	Sasebo (33°10′50″N, 129°38′47″E), Japan	-	-	-	TNS 753674	AB436935
	Hasama (34°58′31″N, 139°46′59″E), Japan	-	-	-	TNS 753675	AB436935

ITS sequences of *H*. *nipponica* from Korean waters have been registered to GenBank in the present study. Data on *H*. *nipponica* from Japanese waters were obtained from the NCBI/GenBank database [15]. The herbarium specimens of Korean *H*. *nipponica* have been deposited in the Herbarium of Kyungpook National University.

### DNA extraction, PCR, and sequencing

Dried leaf tissue was ground in liquid nitrogen, and then genomic DNA was extracted using a DNeasy plant mini-kit (Qiagen, Valencia, USA), following the manufacturer’s protocol. DNA extraction was checked using 1.5% agarose gel electrophoresis followed by ethidium bromide staining. Concentrations of genomic DNA were quantified using a NanoDrop (ND-1000) spectrophotometer.

Internal transcribed spacer (ITS) sequences in the nuclear ribosomal DNA (nrDNA), ITS-1, 5.8S nrDNA, and ITS-2, were amplified using the primer pairs ITS-1 (forward) and ITS-4 (reverse). All PCR reactions were performed using a PTC-100 thermal cycler (Bio-Rad, USA). The amplifications were done using QIAGEN *Taq* polymerase mixed manually with 10× PCR buffer, MgCl_2_, and dNTPs. The thermal cycling conditions were composed of an initial denaturation step at 94°C for 2 min, then 35 cycles at 94°C for 1 min, 50°C for 1 min, 72°C for 2 min, and a final extension time of 10 min at 72°C. The annealing process was conducted for 1 min at 50°C. PCR products were separated by 1.5% agarose gel electrophoresis followed by staining with ethidium bromide. Bands were excised from the agarose gel and purified using a QIAQuick Gel Purification kit (QIAGEN). DNA sequencing reactions were performed using ABI BigDye Terminator v3.1 cycle sequencing kits following the manufacturer’s protocol. DNA sequences were obtained from an ABI 3730xl DNA analyzer.

### Molecular analysis of ITS sequences of *Halophila* species

ITS sequences of *Halophila* species obtained in this study have been submitted to GenBank (http://www.ncbi.nlm.nih.gov/genbank/), and accession numbers were presented in Table 1 and S1 Table. Nucleotide sequence analyses were performed using BioEdit (Ver. 7.1.3) software for sequence compilation and alignment. Gaps were treated as missing data. Additional ITS sequences of *H*. *nipponica* from Japan and other *Halophila* species were obtained from the NCBI/GenBank database and included in the alignment (S1 Table). Approximately 130 ITS sequences of *Halophila* species were retrieved from the NCBI/GenBank database. Identical ITS sequences of the same species at adjacent geographical locations were excluded, and the remaining 47 ITS sequences were included in the alignment to analyze phylogenetic relationships among species of the genus *Halophila*.

A maximum parsimony (MP) tree of ITS regions was obtained using the MEGA 5.1 program [28]. Neighbor-joining (NJ) analysis was performed using the maximum composite likelihood model with 1000 bootstrap replications [29]. The topologies of the phylogenetic trees from the MP and NJ analyses were almost identical, except for differences in bootstrap support values at some nodes. Thus, phylogenetic results from only the MP analysis are presented in this study. In the MP analysis, heuristic searches were performed with the Tree-Bisection-Reconnection (TBR) branch-swapping algorithm. Support for the nodes of the MP tree was determined by calculating bootstrap values based on 1000 replications. Mean similarities between clades, and between species within clades, were calculated using the Kimura 2-parameter model and the numbers of sequence differences were counted using the estimation of pairwise distance model in the MEGA 5.1 program [30].

Relative divergence times of the clades in section *Halophila*, in which all species have a pair of petiolate leaves at each rhizome node, were estimated based on ITS sequence divergence to understand the evolutionary trend of *H*. *nipponica* using NETWORK 4.6 (http://www.fluxus-engineering.com/sharenet.htm) software. Relative divergence times of morphologically similar *Halophila* species to *H*. *nipponica* such as *H*. *okinawensis*, *H*. *gaudichaudii*, and *H*. *ovalis* were also estimated based on ITS sequence divergence. To obtain estimates of the timing of divergence among *Halophila* species based on ITS sequences, we employed a consensus approach for ITS sequence diversity. Values between 1.72 × 10^−9^ and 1.71 × 10^−8^ mutations/site/year, which have been used as the range for ITS mutation rates in herbaceous plants, were employed as nucleotide mutation rates [31,32].

## Results

### Phylogenetic position of *Halophila nipponica*

**Fig 2:**
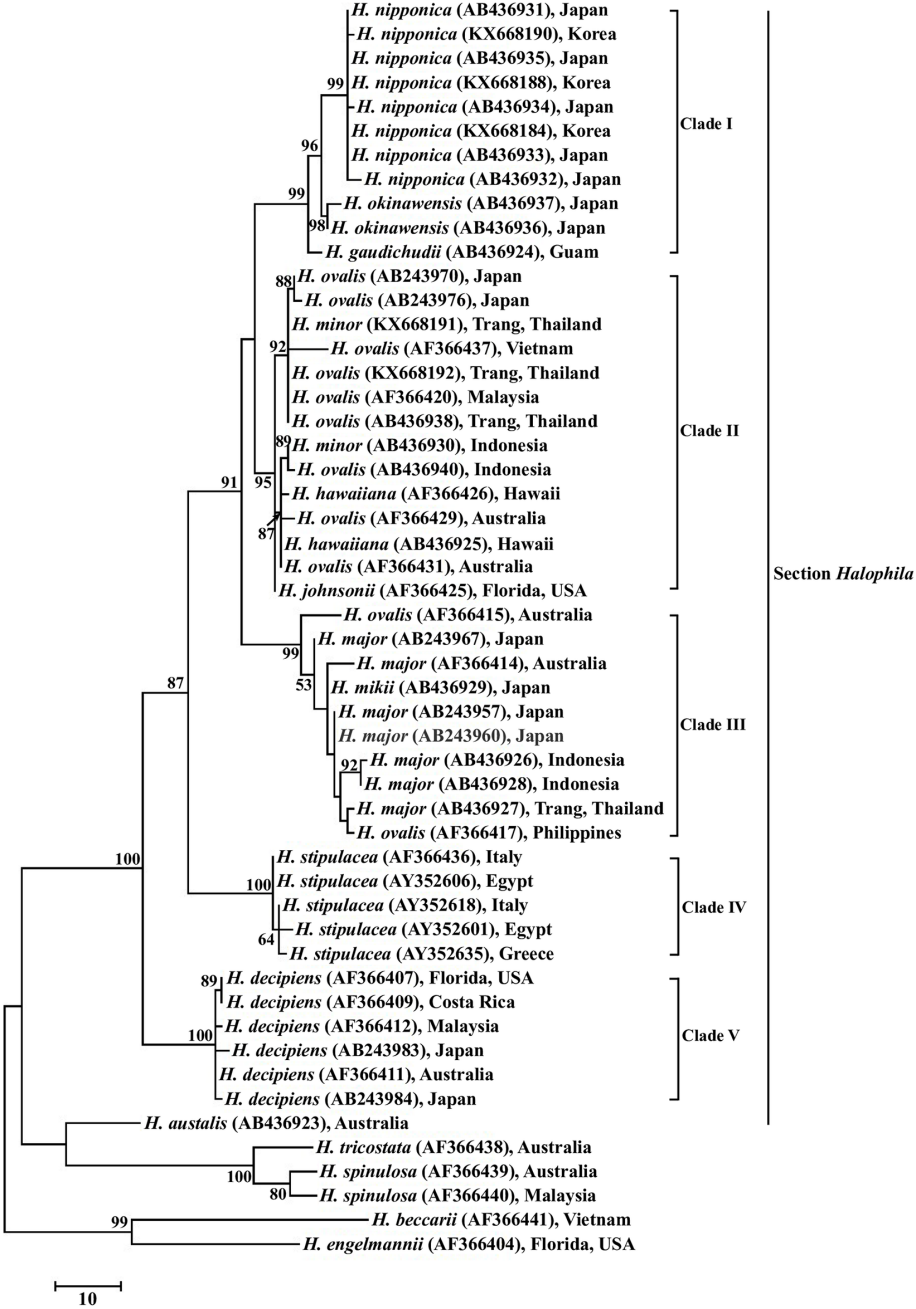
Phylogenetic tree of *Halophila* species inferred from maximum parsimony analysis using 655 base pairs of nrDNA including ITS1, 5.8S rDNA, and ITS2. Bootstrap support values above 50% are shown on branches.

*Halophila* species in section *Halophila* were separated into five well-supported clades with 87–100% bootstrap support in the MP analysis (Fig 2). *H*. *nipponica* was grouped with *H*. *okinawensis* and *H*. *gaudichaudii* from subtropical regions into Clade I of section *Halophila* with 99% bootstrap support (Fig 2). In Clade I, *H*. *nipponica* and *H*. *okinawensis* were separated from *H*. *gaudichaudii*, and all *H*. *nipponica* samples from the Korean and Japanese coastal waters were included in a subgroup of Clade I (Fig 2). Clade II of section *Halophila* was a well-supported group (95% bootstrap value) including *H*. *ovalis*, *H*. *hawaiiana*, *H*. *johnsonii*, and *H*. *minor* (Fig 2). Species within Clade II were further split into two subgroups, with *H*. *johnsonii* falling outside these subgroups. One subgroup included *H*. *minor* from Thailand and *H*. *ovalis* from Japan, Malaysia, Vietnam, and Thailand. The other subgroup consisted of *H*. *hawaiiana* from Hawaii, *H*. *minor* from Indonesia, and *H*. *ovalis* from Australia and Indonesia. *H*. *minor* and *H*. *ovalis* within Clade II were separated by geographical location rather than by species, with one group from Japan, Thailand, Malaysia, and Vietnam and the other group from Indonesia and Australia. Clade III, which received 99% bootstrap support in the MP analysis, consisted of *H*. *major* (previously known as *H*. *euphlebia*), *H*. *mikii* from Japan, *H*. *australis* from Australia, and *H*. *ovalis* from Australia and the Philippines (Fig 2). *H*. *decipiens* and *H*. *stipulacea* formed well-supported monophyletic groups, which received 100% bootstrap support in the MP analysis (Fig 2). Clade IV consisted of the single species, *H*. *stipulacea*, and Clade V consisted of the single species, *H*. *decipiens*. Although *H*. *decipiens* has a wide distributional range in tropical and subtropical waters, separation by geographical location was not supported by ITS sequence analysis (Fig 2).

*Halophila* species which have complex phyllotaxy (*H*. *engelmannii*, *H*. *beccarii*, *H*. *spinulosa*, and *H*. *tricostata*) were clearly separated from species with simple phyllotaxy in section *Halophila*, except for one sample of *H*. *australis* (AB436923), in the phylogenetic tree (Fig 2). *H*. *spinulosa* and *H*. *tricostata* were grouped with 100% bootstrap support in the MP analysis and the group of *H*. *beccarii* and *H*. *engelmannii* was also supported well, with a bootstrap value of 99% (Fig 2).

### ITS sequence similarity of *Halophila nipponica*

Similarities of ITS sequences among the five clades of section *Halophila* ranged from 91.7 to 95.9% (Table 2). Clade I, which includes *H*. *nipponica*, had the greatest similarity to Clade II, which includes *H*. *ovalis*, and the lowest similarity to Clade IV, which consists of a single species, *H*. *stipulacea* (Table 2). Divergence of ITS sequences among species in section *Halophila* occurred primarily in the ITS-1 and ITS-2 regions, while only three polymorphic sites were found in the 5.8S region among the species analyzed (S1 Fig). Among the *Halophila* species in Clade I (*H*. *nipponica*, *H*. *okinawensis*, and *H*. *gaudichaudii*), five and nine polymorphic sites, which were mostly C/T mutations, were found in the ITS-1 and ITS-2 regions, respectively (S1 Fig).

**Table 2:** Similarities (%) and number of ITS sequence differences among five clades in section *Halophila*.

	**Clade I**	**Clade II**	**Clade III**	**Clade IV**	**Clade V**
**Clade I**	-	23.4	33.5	45.7	43.0
**Clade II**	95.9	-	25.6	36.3	37.0
**Clade III**	94.0	95.5	-	40.8	44.8
**Clade IV**	91.7	93.4	92.6	-	35.8
**Clade V**	92.2	93.3	91.8	93.5	-

Values above the dashed diagonal represent the number of ITS sequence differences, while those below the diagonal represent similarities among the five clades of section *Halophila*.

**Fig 3:**
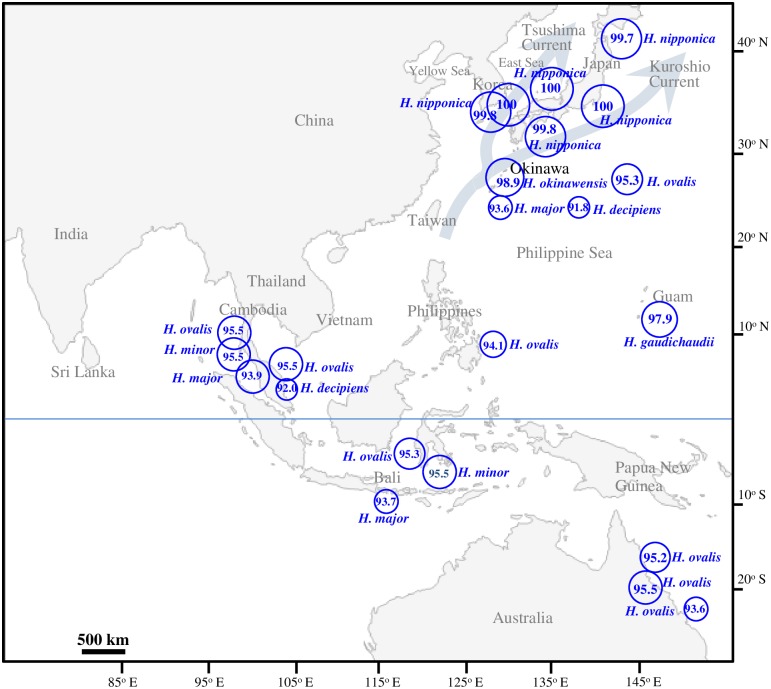
Similarity of ITS sequences between *Halophila nipponica* and other *Halophila* species distributed in the Indo-Pacific and western Pacific regions.

ITS sequences of *H*. *nipponica* from various locations in Korea and Japan were identical or showed less than 0.5% sequence divergence (3-bp difference) (Fig 3; S2 Table). ITS sequences of *H*. *nipponica* showed 1.1–1.6% sequence divergence (98.4–98.9% sequence similarity; 7–10-bp difference) from those of *H*. *okinawensis* from Japan and 2.1–2.5% sequence divergence (97.6–97.9% similarity; 13–15-bp difference) from that of *H*. *gaudichaudii* from Guam (Fig 3; S2 Table). *H*. *nipponica* also showed relatively high sequence similarity in the ITS region to *Halophila* species in Clade II (S2 Table). *H*. *nipponica* showed ITS sequence similarity of 95.2–95.5% to *H*. *minor*, 95.0–95.5% to *H*. *hawaiiana*, and 95.7–96.0% to *H*. *johnsonii* (S2 Table). ITS sequences of *H*. *nipponica* usually showed higher than 94.9% sequence similarity to those of *H*. *ovalis*, except *H*. *ovalis* from Vietnam in Clade II and from Australia and Philippines in Clade III (Fig 3; S2 Table). *H*. *major* from the tropical/subtropical Indo-Pacific showed ITS sequence similarity of 93.6–93.9% to *H*. *nipponica*. However, *H*. *decipiens* from the tropical/subtropical Indo-Pacific in Clade V showed relatively low ITS sequence similarity (91.8–92.0%) to *H*. *nipponica* (Fig 3).

### Relative divergence times of *Halophila* species

**Fig 4:**
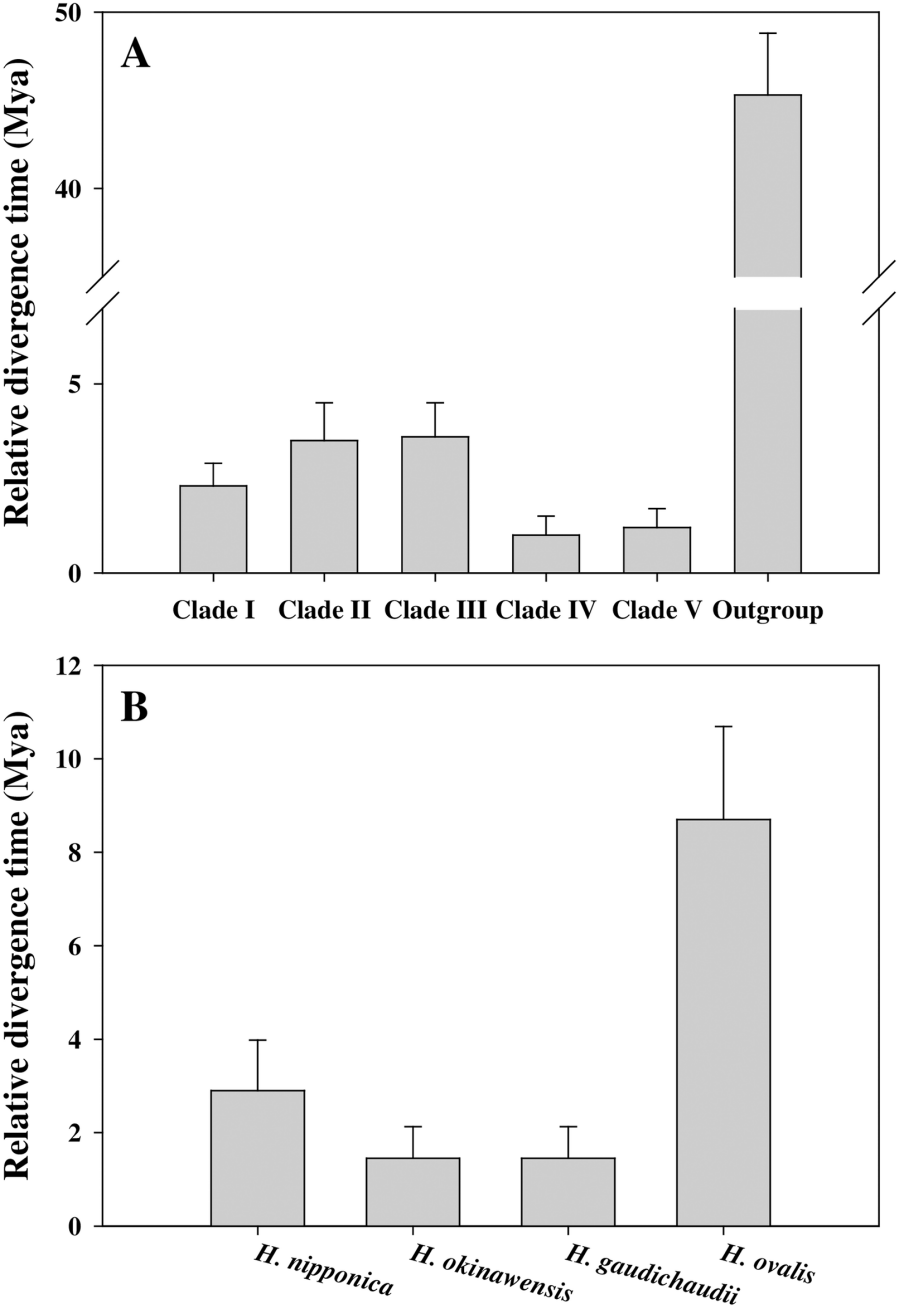
Relative divergence time estimates of five clades in the section *Halophila* (A), and the *Halophila* species (*H*. *nipponica*, *H*. *okinawensis*, and *H*. *gaudichaudii*) in Clade I and *H*. *ovalis* in Clade II (B). Relative divergence time was estimated by ITS sequence diversity using the NETWORK 4.6 program. Values are mean ± SD.

Clades IV and V, which each consisted of a single species, showed more recent relative divergence times than Clades I, II, and III, which each consisted of several *Halophila* species (Fig 4A). Relative divergence times for Clade I, including *H*. *nipponica*, and Clade II, including *H*. *ovalis*, were 2.3 ± 0.6 and 3.5 ± 1.0 Mya, respectively (Fig 4A). *H*. *okinawensis* and *H*. *gaudichaudii* were the youngest species (1.5 ± 0.68 Mya) in Clade I (Fig 4B). Divergence times for *H*. *nipponica* and *H*. *ovalis* were 2.9 ± 1.08 and 8.7 ± 1.99 Mya, respectively (Fig 4B).

## Discussion

### Genetic variability in *Halophila nipponica*

Populations of *H*. *nipponica* have been reported only in the warm temperate coastal waters of Korea and Japan [10,14–16]. When *H*. *nipponica* was described as a new species, this species was considered endemic to Japan [14]. Recently, many *H*. *nipponica* meadows have been observed in the coastal waters of Korea [16,17], but no studies on taxonomic or genetic similarities have been conducted between the populations of *H*. *nipponica* in Japan and Korea. In the present study, ITS sequences of *H*. *nipponica* plants from various locations across its geographic range in the coastal waters of Korea and Japan were identical or showed very low divergence (less than 3-bp difference). Divergence of ITS sequences within *H*. *nipponica* is much lower than the interspecific divergence (3.0–28.5%; 18–172-bp difference) found among other *Halophila* species [15,19,25]. Additionally, all *H*. *nipponica* collections from various locations in Korea and Japan were included in a group, and well separated from other *Halophila* species in the MP analysis. These phylogenetic results using ITS sequences suggest that *H*. *nipponica* is distinct from other *Halophila* species. Additionally, *H*. *nipponica* from Korea and Japan are confirmed to be the same species, and have nearly identical ITS sequences (0–3-bp difference).

In this study, *Halophila* species in the section *Halophila* were clearly separated from the species in other sections by molecular phylogenetic analysis of ITS sequences, and were grouped into five monophyletic clades. Historically, *H*. *nipponica*, *H*. *okinawensis*, and *H*. *gaudichaudii* were described morphologically as separate species on the basis of leaf dimensions [14]. Subsequently, *H*. *nipponica* grouped with *H*. *okinawensis* and *H*. *gaudichaudii* because of relatively high ITS sequence similarity and these three seagrasses were considered conspecific [15]. However, in our study of these species, including new samples collected throughout its current known range, *H*. *nipponica* has higher genetic similarity among all samples (≤ 3-bp) than it does to either *H*. *okinawensis* (7–10-bp) or *H*. *gaudichaudii* (13–15-bp) (S2 Table). ITS sequence divergence of 0–9-bp (0–1.45%) has been considered the level of intraspecific variation for *Halophila* species [15,25,33]. Additionally, *H*. *nipponica*, *H*. *okinawensis*, and *H*. *gaudichaudii* were well separated into 3 groups in the MP analysis. Thus, these 3 *Halophila* species should be considered distinct taxa at either the specific or subspecific level.

*H*. *okinawensis* and *H*. *gaudichaudii* occur in the subtropical region of the western Pacific, whereas *H*. *nipponica* occurs only in the warm temperate region of the northwestern Pacific [14–16]. *H*. *okinawensis* and *H*. *gaudichaudii* are located in the intermediate region between the tropical Indo-Pacific where tropical *Halophila* species occur and the temperate northwestern Pacific where *H*. *nipponica* occurs. Thus, these subtropical *Halophila* species appear to have spread from the tropical Indo-Pacific region due to the influence of the warm Kuroshio Current [14,34].

### Evolutionary trend of *Halophila nipponica*

*Halophila* species in Clade I (*H*. *nipponica*, *H*. *okinawensis*, and *H*. *gaudichaudii*) showed the highest ITS sequence similarity with the tropical/subtropical *Halophila* species in Clade II such as *H*. *ovalis*, *H*. *minor*, *H*. *hawaiiana*, and *H*. *johnsonii*. *H*. *hawaiiana*, and *H*. *johnsonii* in Clade II occur only in the restricted areas and could not be distinguished from *H*. *ovalis* according to many molecular approaches to the identification of *Halophila* species [20,21]. Thus, we suggest that the temperate and subtropical *Halophila* species in Clade I probably have diverged from the tropical *H*. *ovalis* in Clade II. The trend of ITS sequence similarities among the tropical *H*. *ovalis*, the subtropical *H*. *okinawensis* and *H*. *gaudichaudii*, and the temperate *H*. *nipponica* was well matched with the geographical distributions of these *Halophila* species (Fig 3). *Halophila* species, which are more closely distributed geographically with *H*. *nipponica*, usually showed higher genetic similarity with this species. These four *Halophila* species from the tropical, subtropical, and temperate regions are quite similar morphologically [14,15], but we found *H*. *nipponica* to be genetically distinct from *H*. *ovalis* as well as from *H*. *gaudichaudii* and *H*. *okinawensis* according to the ITS sequence analysis.

Although *H*. *nipponica* is distributed in the Temperate North Pacific Bioregion in which the temperate seagrass genera *Zostera*, *Phyllospadix*, and *Ruppia* are dominant, it occurs primarily near the boundary of the Tropical Indo-Pacific Bioregion in which *Halophila* species are common [9]. Thus, propagules of *Halophila* species in the tropical/subtropical Indo-Pacific may travel easily to the temperate coastal waters of Korea and Japan via the warm Kuroshio Current. *H*. *nipponica* is quite similar morphologically to *H*. *ovalis*, and this species was treated as *H*. *ovalis* previously [14,19]. Among the *Halophila* species in the tropical Indo-Pacific, *H*. *ovalis* appears to be the most similar species to *H*. *nipponica* both morphologically and genetically [14,15]. Based on phylogenetic analysis and latitudinal distribution, *Halophila* species (*H*. *nipponica*, *H*. *okinawensis*, and *H*. *gaudichaudii*) within Clade I appear to have diverged from *H*. *ovalis* in tropical Indo-Pacific waters.

Because *H*. *okinawensis* and *H*. *gaudichaudii* occur in subtropical regions, these species might be expected to be intermediate species between the tropical *H*. *ovalis* and the temperate *H*. *nipponica* from an evolutionary perspective. However, according to divergence time estimates, *H*. *nipponica* diverged from *H*. *ovalis* earlier (2.9 Mya) than *H*. *okinawensis* and *H*. *gaudichaudii* (1.5 Mya). Thus, the subtropical *H*. *okinawensis* and *H*. *gaudichaudii* are younger species than the temperate *H*. *nipponica*. This result suggests that the temperate *H*. *nipponica* have not diverged from the subtropical *Halophila* species and the temperate *H*. *nipponica* and the subtropical *H*. *okinawensis* and *H*. *gaudichaudii* may have different evolutionary histories. There is limited information available on ITS sequences of *H*. *okinawensis* and *H*. *gaudichaudii*, which causes difficulty in the accurate estimation of the divergence times among these species. Thus, further genetic studies of *H*. *nipponica*, *H*. *okinawensis*, and *H*. *gaudichaudii* are required to better understand the evolutionary relationships between these *Halophila* species. Recently, *rbc*L and *mat*K sequences have been used for the identification of the common and widespread *Halophila* species such as *H*. *ovalis* and *H*. *decipiens* [35–37]. Analysis of these additional genetic sequences will provide invaluable information on the species identification and evolution of *Halophila* species.

In conclusion, *H*. *nipponica* plants from the various locations in temperate coastal waters of the northwestern Pacific were nearly genetically identical based on ITS sequences. *H*. *nipponica* from the temperate regions of Korea and Japan was grouped with *H*. *okinawensis* and *H*. *gaudichaudii* from the subtropical regions of the western Pacific in Clade I. These temperate and subtropical *Halophila* species in Clade I showed high ITS sequence similarity to the tropical *H*. *ovalis* in Clade II. Based on geographical distribution and similarities in genetics and morphology, *H*. *nipponica* is suggested to have diverged from a tropical *Halophila* species such as *H*. *ovalis*, which was transported from the tropical Indo-Pacific via Pacific Ocean circulation and then adapted to warm temperate environments. According to divergence time estimates, the temperate *H*. *nipponica* was considered an older species than the subtropical *H*. *okinawensis* and *H*. *gaudichaudii* and may have a different evolutionary history with the subtropical *Halophila* species.

## Supporting information

S1 Fig ITS sequence alignments of *Halophila* species within section *Halophila*. The ITS region is composed of the ITS1 (1–225 bp), 5.8S (226–387 bp), and ITS2 (388–631 bp) regions. In ITS sequences of *Halophila* species within the section *Halophila*, the major sequence differences occurred in the ITS1 and ITS2 regions, whereas few sequence differences were found in the 5.8S region.
(DOCX)

S1 Table Geographical locations and GenBank accession numbers of *Halophila* species used for phylogenetic analysis. * ITS sequences of KX668184, KX668185, KX668186, KX668187 and KX668189 were identical.
(DOC)

S2 Table Similarities (%) and the number of differences in ITS sequences among *Halophila* species within Clade I (box) and Clade II. Bold numbers indicate the species *H*. *nipponica*. Values above the dashed diagonal represent the number of ITS sequence differences, while those below the diagonal represent similarities among *Halophila* species in Clade I and Clade II.
(DOC)
