# Supplementary material for: Genetic identification and evolutionary trends of the seagrass Halophila nipponica in temperate coastal waters of Korea
Source: PLoS One. 2017 May 15;12(5):e0177772. doi: 10.1371/journal.pone.0177772 (PMC5432184; doi:10.1371/journal.pone.0177772)
Supplement: S1 Fig — The ITS region is composed of the ITS1 (1–225 bp), 5.8S (226–387 bp), and ITS2 (388–631 bp) regions. In ITS sequences of Halophila species within the section Halophila, the major sequence differences occurred in the ITS1 and ITS2 regions, whereas few sequence differences were found in the 5.8S region. (DOCX) [file pone.0177772.s001.docx]

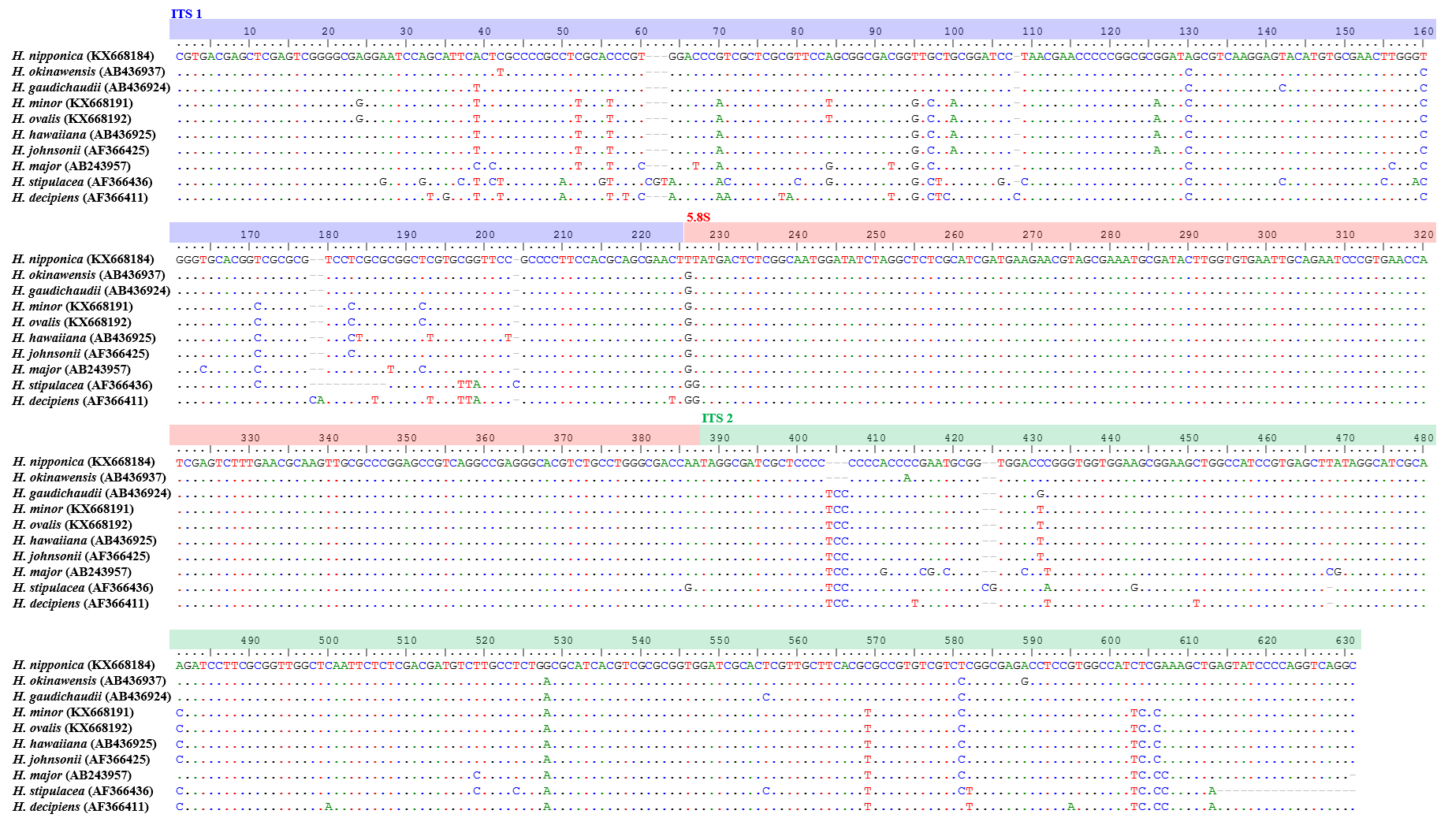


**S1 Figure. ITS sequence alignments of Halophila species within section Halophila.** The ITS region is composed of the ITS1 (1–225 bp), 5.8S (226–387 bp), and ITS2 (388–631 bp) regions. In ITS sequences of *Halophila* species within the section *Halophila*, the major sequence differences occurred in the ITS1 and ITS2 regions, whereas few sequence differences were found in the 5.8S region.
