# Supplementary material for: Genetic identification and evolutionary trends of the seagrass Halophila nipponica in temperate coastal waters of Korea
Source: PLoS One. 2017 May 15;12(5):e0177772. doi: 10.1371/journal.pone.0177772 (PMC5432184; doi:10.1371/journal.pone.0177772)
Supplement: S1 Table — * ITS sequences of KX668184, KX668185, KX668186, KX668187 and KX668189 were identical. (DOC) [file pone.0177772.s002.doc]

**S1 Table. Geographical locations and GenBank accession numbers of *Halophila*** species used for phylogenetic analysis.

| **No.** | **Species** | **Geographical location** | **GenBank**  **accession no.** |
| --- | --- | --- | --- |
| 1 | *Halophila nipponica* | Kanagawa Pref., Japan (35°13′N, 139°37′E) | AB436931 |
| 2 |  | Aomori Pref., Japan (41°00′N, 140°40′E) | AB436932 |
| 3 |  | Yamaguchi Pref., Japan | AB436933 |
| 4 |  | Tokushima Pref., Japan (33°38′N, 134°29′E) | AB436934 |
| 5 |  | Shimane Pref., Japan (36°01′N, 133°01′E) | AB436935 |
| 6* |  | An-do Island, Korea (HN01–HN20)  Sorok-do Island, Korea (HN21–HN28)  Namhae Island, Korea (HN31–HN42)  Koje Island, Korea (HN51–55, HN58–60)  Geomun-do Island, Korea (HN61, 63, 64) | KX668184  KX668185  KX668186  KX668187  KX668189 |
| 7 |  | Koje Island, Korea (HN56, 57) | KX668188 |
| 8 |  | Geomun-do Island, Korea (HN62) | KX668190 |
| 9 | *Halophila okinawensis* | Okinawa, Japan | AB436936 |
| 10 |  | Iriomote Island, Japan | AB436937 |
| 11 | *Halophila gaudichaudii* | Agania Bay, Guam | AB436924 |
| 12 | *Halophila ovalis* | Okinawa, Japan (26°17′14.3″N, 127°52′14.5″E) | AB243970 |
| 13 |  | Okinawa, Japan (24°19′21.6″N, 124°04′1.02″E) | AB 243976 |
| 14 |  | Trang, Thailand | AB436938 |
| 15 |  | Flores Island, Indonesia | AB436940 |
| 16 |  | Shoalwater Bay, Australia | AF366415 |
| 17 |  | Makadong, Philippines | AF366417 |
| 18 |  | Kuala Setiu, Malaysia | AF366420 |
| 19 |  | Cape Cleveland, Australia | AF366429 |
| 20 |  | Dingo Beach, Australia | AF366431 |
| 21 |  | Gia Luan, Vietnam | AF366437 |
| 22 |  | Trang, Thailand (HO) | KX668192 |
| 23 | *Halophila minor* | Flores Island, Indonesia | AB436930 |
| 24 |  | Trang, Thailand (HM) | KX668191 |
| 25 | *Halophila hawaiiana* | Hawaii, USA | AB436925 |
| 26 |  | Hawaii, USA | AF366426 |
| 27 | *Halophila johnsonii* | Florida, USA | AF366425 |
| 28 | *Halophila major* | Tokusima Pref., Japan (33°32′41″N, 134°18′53″E) | AB243957 |
| 29 |  | Okinawa, Japan (24°29′13.2″N, 124°13′47.6″E) | AB243960 |
| 30 |  | Okinawa, Japan (26°17′56.1″N, 127°50′31.7″E) | AB243967 |
| 31 |  | Sumbawa, Indonesia | AB436926 |
| 32 |  | Trang, Thailand | AB436927 |
| 33 |  | Bali, Indonesia | AB436928 |
| 34 | *Halophila mikii* | Kagoshima Pref., Japan | AB436929 |
| 35 | *Halophila stipulacea* | Sicily, Italy | AF366436 |
| 36 |  | Small Creek, Egypt | AY352601 |
| 37 |  | Ras Mohammed, Egypt | AY352606 |
| 38 |  | Vulcano Island, Italy | AY352618 |
| 39 |  | Rhodes Island, Greece | AY352635 |
| 40 | *Halophila decipiens* | Okinawa, Japan (26°18′50.8″N, 127°51′50.3″E) | AB243983 |
| 41 |  | Okinawa, Japan (26°31′24.9″N, 128°09′11.4″E) | AB243984 |
| 42 |  | Florida, USA | AF366407 |
| 43 |  | Southeastern Costa Rica | AF366409 |
| 44 |  | Dunk Island, Australia | AF366411 |
| 45 |  | Kuala Setiu, Malaysia | AF366412 |
| 46 | *Halophila australis* | Victoria, Australia | AB436923 |
| 47 |  | Two Peoples Bay, Australia | AF366414 |
| 48 | *Halophila spinulosa* | Whitsunday Island, Australia | AF366439 |
| 49 |  | Pulau Perhentian, Malaysia | AF366440 |
| 50 | *Halophila beccarii* | Gia Luan, Vietnam | AF366441 |
| 51 | *Halophila engelmannii* | Florida Bay, USA | AF366404 |
| 52 | *Halophila tricostata* | Whitsunday Island, Australia | AF366438 |

* ITS sequences of KX668184, KX668185, KX668186, KX668187 and KX668189 were identical.
