# Supplementary material for: Genetic identification and evolutionary trends of the seagrass Halophila nipponica in temperate coastal waters of Korea
Source: PLoS One. 2017 May 15;12(5):e0177772. doi: 10.1371/journal.pone.0177772 (PMC5432184; doi:10.1371/journal.pone.0177772)
Supplement: S2 Table — Bold numbers indicate the species H. nipponica. Values above the dashed diagonal represent the number of ITS sequence differences, while those below the diagonal represent similarities among Halophila species in Clade I and Clade II. (DOC) [file pone.0177772.s003.doc]

**S2 Table. Similarities (%) and the number of differences in ITS sequences among *Halophila*** species within Clade I (box) and Clade II.

| Sample No. | 1 | 2 | 3 | 4 | 5 | 6 | 7 | 8 | 9 | 10 | 11 | 12 | 13 | 14 | 15 | 18 | 19 | 20 | 21 | 22 | 23 | 24 | 25 | 26 | 27 |
| --- | --- | --- | --- | --- | --- | --- | --- | --- | --- | --- | --- | --- | --- | --- | --- | --- | --- | --- | --- | --- | --- | --- | --- | --- | --- |
| 1 *H. nipponica*, Japan | **-** | **2** | **0** | **1** | **0** | **0** | **1** | **1** | 7 | 8 | 13 | 29 | 31 | 28 | 29 | 28 | 30 | 28 | 41 | 28 | 28 | 28 | 28 | 29 | 25 |
| 2 *H. nipponica*, Japan | **99.7** | **-** | **2** | **3** | **2** | **2** | **3** | **3** | 9 | 10 | 15 | 31 | 33 | 30 | 31 | 30 | 32 | 30 | 43 | 30 | 30 | 30 | 30 | 31 | 27 |
| 3 *H. nipponica*, Japan | **100** | **99.7** | **-** | **1** | **0** | **0** | **1** | **1** | 7 | 8 | 13 | 29 | 31 | 28 | 29 | 28 | 30 | 28 | 41 | 28 | 28 | 28 | 28 | 29 | 25 |
| 4 *H. nipponica*, Japan | **99.8** | **99.5** | **99.8** | **-** | **1** | **1** | **2** | **2** | 8 | 9 | 14 | 30 | 32 | 29 | 30 | 29 | 31 | 29 | 42 | 29 | 29 | 29 | 29 | 30 | 26 |
| 5 *H. nipponica*, Japan | **100** | **99.7** | **100** | **99.8** | **-** | **0** | **1** | **1** | 7 | 8 | 13 | 29 | 31 | 28 | 29 | 28 | 30 | 28 | 41 | 28 | 28 | 28 | 28 | 29 | 25 |
| 6 *H. nipponica*, Korea | **100** | **99.7** | **100** | **99.8** | **100** | **-** | **1** | **1** | 7 | 8 | 13 | 29 | 31 | 28 | 29 | 28 | 30 | 28 | 41 | 28 | 28 | 28 | 28 | 29 | 25 |
| 7 *H. nipponica*, Korea | **99.8** | **99.5** | **99.8** | **99.7** | **99.8** | **99.8** | **-** | **2** | 8 | 9 | 14 | 30 | 32 | 29 | 30 | 29 | 31 | 29 | 42 | 29 | 29 | 29 | 29 | 30 | 26 |
| 8 *H. nipponica*, Korea | **99.8** | **99.5** | **99.8** | **99.7** | **99.8** | **99.8** | **99.7** | **-** | 8 | 9 | 14 | 30 | 32 | 29 | 30 | 29 | 31 | 29 | 42 | 29 | 29 | 29 | 29 | 30 | 26 |
| 9 *H. okinawensis*, Japan | 98.9 | 98.5 | 98.9 | 98.7 | 98.9 | 98.9 | 98.7 | 98.7 | - | 3 | 8 | 24 | 26 | 23 | 24 | 23 | 25 | 23 | 36 | 23 | 23 | 23 | 23 | 24 | 20 |
| 10 *H. okinawensis*, Japan | 98.7 | 98.4 | 98.7 | 98.5 | 98.7 | 98.7 | 98.5 | 98.5 | 99.5 | - | 11 | 27 | 29 | 26 | 27 | 26 | 28 | 26 | 39 | 26 | 26 | 26 | 26 | 27 | 23 |
| 11 *H. gaudichaudii*, Guam | 97.9 | 97.6 | 97.9 | 97.7 | 97.9 | 97.9 | 97.9 | 97.7 | 98.7 | 98.2 | - | 21 | 23 | 20 | 21 | 20 | 22 | 20 | 34 | 20 | 20 | 20 | 20 | 21 | 17 |
| 12 *H. ovalis*, Japan | 95.3 | 95.0 | 95.3 | 95.2 | 95.3 | 95.3 | 95.2 | 95.2 | 96.1 | 95.6 | 96.6 | - | 2 | 2 | 8 | 2 | 9 | 7 | 16 | 2 | 7 | 2 | 7 | 8 | 4 |
| 13 *H. ovalis*, Japan | 95.1 | 94.7 | 95.1 | 94.9 | 95.1 | 95.1 | 94.9 | 94.9 | 95.8 | 95.3 | 96.3 | 99.7 | - | 4 | 10 | 4 | 11 | 9 | 18 | 4 | 9 | 4 | 9 | 10 | 6 |
| 14 *H. ovalis*, Thailand | 95.5 | 95.2 | 95.5 | 95.3 | 95.5 | 95.5 | 95.3 | 95.3 | 96.3 | 95.8 | 96.8 | 99.7 | 99.4 | - | 7 | 0 | 8 | 6 | 14 | 0 | 6 | 0 | 6 | 7 | 3 |
| 15 *H. ovalis*, Indonesia | 95.3 | 95.0 | 95.3 | 95.2 | 95.3 | 95.3 | 95.2 | 95.2 | 96.1 | 95.7 | 96.6 | 98.7 | 98.4 | 98.9 | - | 7 | 5 | 3 | 21 | 7 | 1 | 7 | 3 | 4 | 4 |
| 18 *H. ovalis*, Malaysia | 95.5 | 95.2 | 95.5 | 95.3 | 95.5 | 95.5 | 95.3 | 95.3 | 96.3 | 95.8 | 96.8 | 99.7 | 99.4 | 100 | 98.9 | - | 8 | 6 | 14 | 0 | 6 | 0 | 6 | 7 | 3 |
| 19 *H. ovalis*, Australia | 95.2 | 94.9 | 95.2 | 95.0 | 95.2 | 95.2 | 95.0 | 95.0 | 96.0 | 95.5 | 96.5 | 98.6 | 98.2 | 98.7 | 99.2 | 98.7 | - | 2 | 22 | 8 | 4 | 8 | 4 | 5 | 5 |
| 20 *H. ovalis*, Australia | 95.5 | 95.2 | 95.5 | 95.3 | 95.5 | 95.5 | 95.3 | 95.3 | 96.3 | 95.8 | 96.8 | 98.9 | 98.6 | 99.0 | 99.5 | 99.0 | 99.7 | - | 20 | 6 | 2 | 6 | 2 | 3 | 3 |
| 21 *H. ovalis*, Vietnam | 93.5 | 93.2 | 93.5 | 93.3 | 93.5 | 93.5 | 93.3 | 93.3 | 94.3 | 93.8 | 94.6 | 97.5 | 97.1 | 97.8 | 96.7 | 97.8 | 96.5 | 96.8 | - | 14 | 20 | 14 | 20 | 21 | 17 |
| 22 *H. ovalis*, Thailand | 95.5 | 95.2 | 95.5 | 95.3 | 95.5 | 95.5 | 95.3 | 95.3 | 96.3 | 95.8 | 96.8 | 99.7 | 99.4 | 100 | 98.9 | 100 | 98.7 | 99.0 | 97.8 | - | 6 | 0 | 6 | 7 | 3 |
| 23 *H. minor*, Indonesia | 95.5 | 95.2 | 95.5 | 95.3 | 95.5 | 95.5 | 95.3 | 95.3 | 96.3 | 95.8 | 96.8 | 98.9 | 98.6 | 99.0 | 99.8 | 99.0 | 99.4 | 99.7 | 96.8 | 99.0 | - | 6 | 2 | 3 | 3 |
| 24 *H. minor*, Thailand | 95.5 | 95.2 | 95.5 | 95.3 | 95.5 | 95.5 | 95.3 | 95.3 | 96.3 | 95.8 | 96.8 | 99.7 | 99.4 | 100 | 98.9 | 100 | 98.7 | 99.0 | 97.8 | 100 | 99.0 | - | 6 | 7 | 3 |
| 25 *H. hawaiiana*, USA | 95.5 | 95.2 | 95.5 | 95.3 | 95.5 | 95.5 | 95.3 | 95.3 | 96.3 | 95.8 | 96.8 | 98.9 | 98.6 | 99.0 | 99.5 | 99.0 | 99.4 | 99.7 | 96.8 | 99.0 | 99.7 | 99.0 | - | 1 | 3 |
| 26 *H. hawaiiana*, USA | 95.3 | 95.0 | 95.3 | 95.2 | 95.3 | 95.3 | 95.2 | 95.2 | 96.1 | 95.7 | 96.6 | 98.7 | 98.4 | 98.9 | 99.4 | 98.9 | 99.2 | 99.5 | 96.7 | 98.9 | 99.5 | 98.9 | 99.8 | - | 4 |
| 27 *H. johnsonii*, USA | 96.0 | 95.7 | 96.0 | 95.8 | 96.0 | 96.0 | 95.8 | 95.8 | 96.8 | 96.3 | 97.3 | 99.4 | 99.0 | 99.5 | 99.4 | 99.5 | 99.2 | 99.5 | 97.3 | 99.5 | 99.5 | 99.5 | 99.5 | 99.4 | - |

Bold numbers indicate the species *H. nipponica*. Values above the dashed diagonal represent the number of ITS sequence differences, while those below the diagonal represent similarities among *Halophila* species in Clade I and Clade II.
